# Patternable Poly(chloro-p-xylylene) Film with Tunable Surface Wettability Prepared by Temperature and Humidity Treatment on a Polydimethylsiloxane/Silica Coating

**DOI:** 10.3390/ma11040486

**Published:** 2018-03-23

**Authors:** Yonglian Yu, Hong Shao, Zhoukun He, Changyu Tang, Jian Yang, Yongsheng Li, Cong Wang, Xiuyun Li, Maobing Shuai, Jun Mei

**Affiliations:** 1 State Key Laboratory Cultivation Base for Nonmetal Composites and Functional Materials, School of Materials Science and Engineering, Southwest University of Science and Technology, Mianyang 621010, China; Yonglian_Yu@163.com; 2 Chengdu Green Energy and Green Manufacturing Technology R&D Center, Chengdu Development Center of Science and Technology, China Academy of Engineering Physics, Chengdu 610207, China; 15882071673@163.com (H.S.); sugarchangyu@163.com (C.T.); m18180544316@163.com (J.Y.); yongsli718@163.com (Y.L.); congwang_polymer@163.com (C.W.); meijun12@126.com (J.M.); 3 Science and Technology on Surface Physics and Chemistry Laboratory, Mianyang 621907, China; shuaimb@sina.com

**Keywords:** Poly(chloro-p-xylylene), surface wettability, polydimethylsiloxane, silica nanoparticles, superhydrophobic coating

## Abstract

Poly(chloro-p-xylylene) (PPXC) film has a water contact angle (WCA) of only about 84°. It is necessary to improve its hydrophobicity to prevent liquid water droplets from corroding or electrically shorting metallic circuits of semiconductor devices, sensors, microelectronics, and so on. Herein, we reported a facile approach to improve its surface hydrophobicity by varying surface pattern structures under different temperature and relative humidity (RH) conditions on a thermal curable polydimethylsiloxane (PDMS) and hydrophobic silica (SiO_2_) nanoparticle coating. Three distinct large-scale surface patterns were obtained mainly depending on the contents of SiO_2_ nanoparticles. The regularity of patterns was mainly controlled by the temperature and RH conditions. By changing the pattern structures, the surface wettability of PPXC film could be improved and its WCA was increased from 84° to 168°, displaying a superhydrophobic state. Meanwhile, it could be observed that water droplets on PPXC film with superhydrophobicity were transited from a “Wenzel” state to a “Cassie” state. The PPXC film with different surface patterns of 200 μm × 200 μm and the improved surface hydrophobicity showed wide application potentials in self-cleaning, electronic engineering, micro-contact printing, cell biology, and tissue engineering.

## 1. Introduction

Poly(chloro-p-xylylene) (PPXC) film has been extensively applied in the fields of semiconductor devices, sensors, microelectronics, material moisture protection, and so on [1,2,3,4]. However, it has weak hydrophobicity with a water contact angle (WCA) of only about 84° [5]. It is necessary to improve its hydrophobicity to prevent liquid water droplets from corroding or electrically shorting metallic circuits of the devices. Superhydrophobic coating [6,7,8,9,10,11,12,13,14] with a WCA greater than 150° and a sliding angle (SA) of less than 10° was used to prevent water droplets from wetting PPXC film. A facile strategy of improving surface hydrophobicity to superhydrophobicity of PPXC film is important in industrial applications of PPXC film [15,16,17]. The surface wettability can be tuned by changing surface morphologies or surface chemical compositions [18,19,20,21,22,23,24,25,26]. Regulation of surface morphologies had been extensively explored for pattern structures [27,28,29,30,31,32,33,34], which could be obtained by printing [35,36], self-assembly of block copolymer [37], and femtosecond laser structuring [38]. However, these methods could not satisfy the requirements of industrial applications due to some key defects, such as the small area of patterns, multi-step processes, and high cost.

Breath figures (BF) based on self-assembled water droplet arrays as dynamic templates had been extensively used to produce ordered honeycomb polymer films. This method had obvious advantages in cost and versatility [39,40,41,42], but the surface morphologies of BF films were closely related to the self-assembled water droplet arrays, which could be tuned by changing solvents, polymers, the concentration of solution, the temperature, and relative humidity (RH) [43,44]. Due to the complexity of these influencing factors, a facile and inexpensive strategy to obtain large-scale patterns on PPXC film still remains a challenge. Controlling the treatment conditions of polymer coating such as temperature and RH was considered as a facile strategy to achieve tunable surface morphology and wettability. However, the effects of temperature and RH on the anti-stick plastic substrates such as PPXC film were seldom reported. Besides, most of previous studies on the BF method were focused on polymers without thermal curing such as polystyrene, and thermal curable polymers such as polydimethylsiloxane (PDMS) used as replica materials were seldom explored [45,46]. Meanwhile, the introduction of hydrophobic silica (SiO_2_) nanoparticles under temperature and RH treatments would have great effects on the surface morphology and surface wettability of PPXC film. Therefore, we herein reported a facile strategy to obtain large-scale patterns of 200 μm × 200 μm and tune surface wettability of PPXC film with a thermal curable PDMS/SiO_2_ coating. The pattern structures obtained by the BF method were usually porous honeycomb structures, but herein three structures (special raised bowl-shaped structures, traditional porous structures, and nanoparticle aggregation structures) were obtained on PPXC film. Moreover, the effects of different treatment conditions such as the content of SiO_2_ nanoparticles, thermal cross-linking temperature of PDMS, and RH on the morphology evolution of PDMS/SiO_2_ coatings were explored systematically, and the surface wettability of PPXC film could be improved from weak hydrophobicity with a WCA of about 84° to superhydrophobicity with a WCA of about 168°. Meanwhile, a typical transition of water droplets from a “Wenzel” state to a “Cassie” state on the surface was observed after tuning the treatment conditions. This transition was demonstrated by the sliding behavior of water droplets on the surface and the temporal changes of WCA on samples. To the best of our knowledge, it is the first time this engineering strategy to obtain large-scale different patterns on PPXC film, without destroying the original PPXC film, has been reported.

## 2. Materials and Methods

### 2.1. Materials

Poly(chloro-p-xylylene) (PPXC) film with a thickness of about 110 μm was kindly provided by China Academy of Engineering Physics. Polydimethylsiloxane adhesive (PDMS, SE1700) and its curing agent (10:1 in weight ratio) were purchased from Dow Corning (Midland, MI, USA). Hydrophobic SiO_2_ nanoparticles (JT-SQ, 10~30 nm) were purchased from Chengdu Today Company (Chengdu, China). Alcohol and hexamethylene were issued by Kelong Chemical Company (Chengdu, China). Deionized water was produced by a water purifier purchased from Thermo Scientific (NANO pure, Waltham, MA, USA) to obtain a certain RH condition.

### 2.2. Dip Coating PDMS/SiO_2_ on PPXC Film

**Figure 1:**
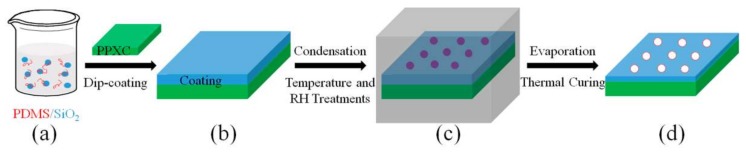
Fabrication procedures of Polydimethylsiloxane (PDMS)/SiO_2_ coating on Poly(chloro-p-xylylene) (PPXC) film controlled by different temperatures and relative humidity (RH) treatment conditions. (**a**) Fabrication of PDMS/SiO_2_ suspension; (**b**) Dip coating of PDMS/SiO_2_ on PPXC film; (**c**) Water droplet condensation on PPXC film in controlled temperature and RH conditions; (**d**) Thermal curing of PDMS/SiO_2_ coating on PPXC film and evaporation of water droplets.

The PDMS with curing agent (10:1 in weight ratio) and SiO_2_ nanoparticles (0.0~2.5 wt % in the whole suspension) were dispersed in hexamethylene under an ultrasonication (100 W) for 30 min to form a homogeneous suspension (40 mg/mL, Figure 1a). The PPXC film was firstly cleaned with alcohol and deionized water three times. After that, it was dip-coated one time in PDMS-based suspension for 5 min by a dip coating machine (SYDC-100, Shanghai SAN-YAN Technology Co., Ltd., Shanghai, China) at a lowering speed of 6000 μm/s and a lifting speed of 1000 μm/s (Figure 1b). Then, the coated PPXC film was treated at different temperatures (60 or 80 °C) and RH (55% or 95%) for 90 min in a constant-temperature and constant-humidity environmental test chamber (Figure 1c, LX/JS01, Shanghai Luxuan Apparatus, Shanghai, China). Finally, samples with different patterns were taken out of the chamber for further characterization after the evaporation of water droplets and the thermal curing of PDMS (Figure 1d).

### 2.3. Characterization

#### 2.3.1. Differential Scanning Calorimetry (DSC) Measurements

A DSC instrument (Mettler Toledo, Zurich, Switzerland) was implemented to observe the thermal curing behavior of PDMS. For the non-isothermal curing measurement, the sample was heated from 30 to 200 °C at a heating rate of 10 °C/min in a nitrogen atmosphere. For the isothermal curing measurement, the sample in the DSC cell was quickly heated to the desired curing temperature and then maintained at that constant temperature for 90 min. For each sample, three replicate scans at different positions were collected to confirm the final results.

#### 2.3.2. Fourier Transform Infrared (FTIR) Measurements

The curing reaction of PDMS on PPXC film was explored by FTIR (Nicolet-iS10, Thermo Fisher Company, Waltham, MA, USA) in the range of 4000–400 cm^−1^ under a transmittance mode. The samples were mixed with KBr powder and then pressed into disks with a thickness of 0.3 mm for FTIR characterization. For each sample, three replicate scans at different positions were tested to confirm the shown value.

#### 2.3.3. Surface Morphology Observation

The surface morphologies of samples were reviewed by scanning electron microscopy (SEM, XL-30-ESEM-FEG instrument, FEI PHILIPS, Brno, Czech Republic) under an acceleration voltage of 20 kV at three different positions. A thin layer of Au was sputter-coated on the film to improve its conductivity before the test. Atomic force microscopy (AFM) images were produced by an atomic force microscope (SPI4000, Seiko Instruments, Chiba, Japan) at ambient conditions in a tapping mode. Commercial silicon nitride tips with the tip radius of about 10 nm were used as received from the indentors in the AFM indentation experiments. Meanwhile, the surface roughness (root-mean-square, RMS) of the samples was the average of five measurements of scan areas of 20 μm × 20 μm.

#### 2.3.4. Contact Angle Measurements

The static water contact angles (WCA) of the samples were carried on a contact angle goniometer (DSA100, Kruss, Hamburg, Germany) with a volume of 5 μL of deionized water droplets on the surface at ambient temperature. The dynamic water droplet behavior was characterized by a sliding angle (SA) of 10 μL of deionized water droplets on the surface at ambient temperature. The WCA and SA mentioned in this study were the averaged value of at least five different positions on each sample.

## 3. Results and Discussion

### 3.1. Effects of Temperature, RH, and Content of SiO_2_ on the Curing Behavior of PDMS

**Figure 2:**
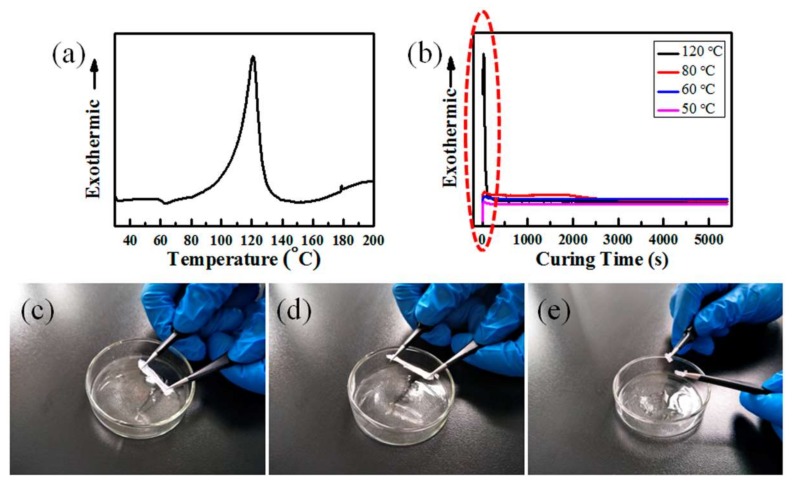
(**a**) Non-isothermal from 30 to 200 °C; (**b**) Isothermal DSC curves for 90 min at different temperatures of PDMS; (**c**–**e**) Screenshots of PDMS during stretching after different curing temperatures at 80, 60, and 50 °C for 90 min under 55% RH.

**Figure 3:**
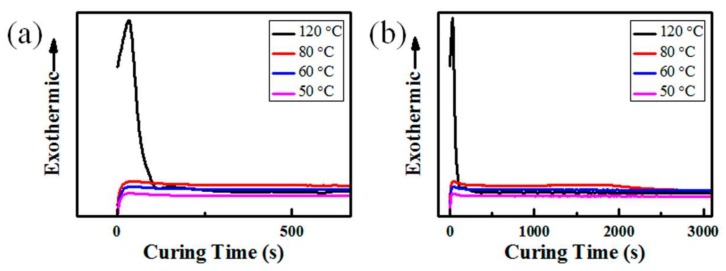
Enlarged images of isothermal differential scanning calorimetry (DSC) curves of PDMS treated at different temperatures. (**a**) Curing time within 500 s; and (**b**) Curing time within 3000 s.

Owing to the thermal curability of PDMS, the temperature is an important factor in the cross-linking reaction process. As shown in Figure 2a, the non-isothermal exothermic curve characterized by DSC indicates that the PDMS curing reaction occurs at about 60 °C and reaches a maximum reaction rate at near 120 °C with the appearance of an exothermic peak. To further investigate the cross-linking reaction behavior of PDMS at different temperatures, isothermal DSC curves of PDMS at 120, 80, 60, and 50 °C for 90 min were determined (Figure 2b). The curing reaction reaches a maximum rate within 40 s at 120 °C with the appearance of an obvious exothermic peak, but only a small exothermic peak occurred within 60 s at 80, 60, or 50 °C (red rectangle frame in Figure 2b and enlarged image in Figure 3a). Therefore, the curing reaction rate was obviously decreased at a lower curing temperature, and the required curing time of PDMS was increased. At 120 °C, the cross-linking reaction ends within 500 s, but it extended to 3000 s at 80, 60, or 50 °C (red rectangle frame in Figure 2b, and enlarged image in Figure 3b). Therefore, the curing reaction time for all samples in this study was set as 90 min to ensure the complete curing of PDMS. Based on the elasticity of cured PDMS, we further investigated whether the PDMS was completely cured or not by a repeated stretching test of the sample after curing at 80, 60, and 50 °C for 90 min under 55% RH (abbreviated as 80 °C-55% RH). The completely cured PDMS with good elasticity after curing at 80 °C and 60 °C could retain its original shape after several stretches (Figure 2c,d). However, the sample cured at 50 °C showed a typical viscous state (Figure 2e) indicating that PDMS was not completely cured. Therefore, the cross-linking reaction temperature of PDMS with a completely cured state for 90 min under 55% RH should not be lower than 60 °C.

**Figure 4:**
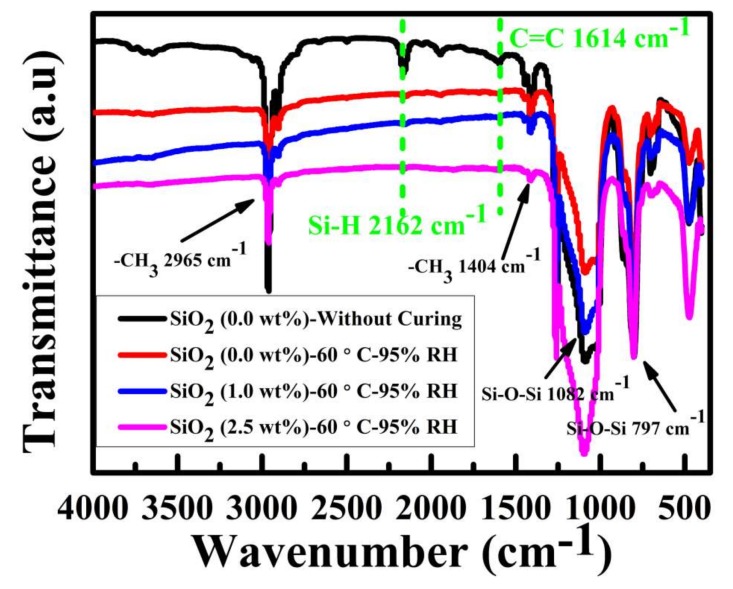
FTIR results of the samples with different contents of SiO_2_ at 60 °C-95% RH treatment.

Furthermore, FTIR results shown in Figure 4 are used to investigate the effects of higher RH (95%) and different contents of SiO_2_ nanoparticles on the cross-linking reaction of PDMS. As for the sample (0.0 wt % of SiO_2_) without curing, the reactive groups of Si–H (2162 cm^−1^) and C=C (1614 cm^−1^) are obviously observed in green dash line. However, there are no typical peaks for these reactive groups after the cross-linking reaction at 60 °C-95% RH, indicating that PDMS can be thermally cured completely under a higher RH compared to a lower RH (55%) shown in Figure 2d. Similarly, the reactive groups of Si–H and C=C also cannot be observed after curing at 60 °C-95% RH for the samples with the increased contents of SiO_2_ nanoparticles (1.0 and 2.5 wt % of SiO_2_). Meanwhile, the characteristic peaks indicated the existence of typical groups in PDMS molecular chains at 2965 cm^−1^, 1404 cm^−1^, 1082 cm^−1^, and 797 cm^−1^ ascribed to -CH_3_ and Si-O-Si groups, respectively. Thus, the higher RH and different contents of SiO_2_ nanoparticles had no obvious effect on the complete thermal curing of PDMS at 60 °C for 90 min. Therefore, the surface morphologies of samples treated for 90 min in the study were in a stable and ultimate state and the surface morphology evolution is discussed below.

### 3.2. Surface Morphology Evolutions of PDMS/SiO_2_ Coating on PPXC Film under Different Treatment Conditions

**Figure 5:**
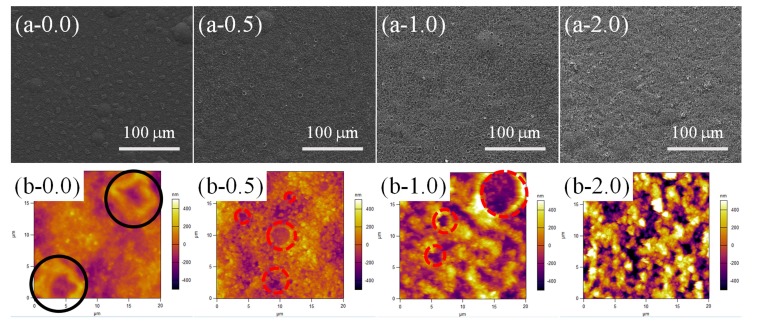
Scanning electron microscopy (SEM) (**a-*x***) and higher magnification atomic force microscopy (AFM) (**b-*x***) images for samples with different contents (***x***) of SiO_2_ at 80 °C-55% RH treatment.

Although PDMS with different contents of SiO_2_ (from 0.0 to 2.5 wt %) could be thermally cured completely at different cross-linking reaction temperatures (120, 80, and 60 °C for 90 min) and different RH treatment conditions (55% and 95%), the cross-linking reaction processes might differ from each other and result in different surface morphologies. SEM and AFM images were implemented to confirm this prediction. Surface morphology evaluation of the samples with different contents of SiO_2_ (0.0, 0.5, 1.0, and 2.0 wt %) treated at 80 °C-55% RH is shown in Figure 5. A large number of special raised bowl-shaped structures (RBS) could be found on the surface of PPXC film with pure PDMS. The special RBS showed the homogeneous distribution on the whole PPXC film (Figure 5(a-0.0)), but a similar phenomenon was not reported in previous studies on PPXC film with different temperatures or RH treatment conditions. Furthermore, a higher magnification of RBS is characterized by the AFM image (black circles in Figure 5(b-0.0). Most of the RBS had a similar typical dimension of about 7 μm in length, and the periphery of RBS was obviously higher than the center of RBS, as indicated by the altitude scale on the right of the AFM image. 

When low contents (0.5 and 1.0 wt %) of SiO_2_ nanoparticles were added in PDMS coating, RBS disappeared and the traditional porous structures (TPS, similar to the pores formed via a traditional “Breath Figures” method) appeared (Figure 5(a-0.5) and (a-1.0). The number of TPS showed no significant variation compared to that of RBS, but the dispersion of TPS is much larger than that of RBS (Figure 5(a-0.0)). Meanwhile, the dimension of TPS becomes inhomogeneous with a distribution between 1 and 7 μm (red dash circles in high-magnification AFM images, Figure 5(b-0.5) and (b-1.0). With a high content (2.0 wt %) of SiO_2_ nanoparticles in PDMS coating (Figure 5(a-2.0)), RBS or TPS did not appear, but a lot of nanoparticle aggregation structures (NAS) occurred, like previous studies [47,48]. The distribution of NAS is very homogeneous, as shown in a high-magnification AFM image (Figure 5(b-2.0)), due to the good dispersion of PDMS and SiO_2_ in hexamethylene solution.

**Figure 6:**
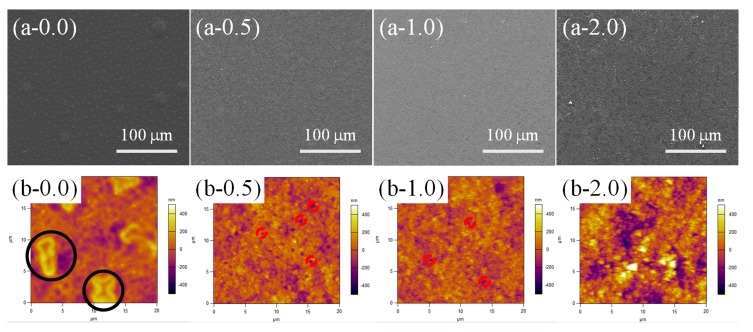
SEM (**a-*x***) and higher magnification AFM (**b-*x***) images for the samples with different contents (***x***) of SiO_2_ at 60 °C-55% RH treatment.

As mentioned above, lower temperature increased the time required for the cross-linking reaction of PDMS. In this section, we further investigated the surface morphology evaluation of the samples with different contents of SiO_2_ (0.0, 0.5, 1.0 and 2.0 wt %) at a lower curing temperature (60 °C for 90 min under 55% RH, Figure 6). A large number of RBS with homogeneous distribution could be found on the whole PPXC film with pure PDMS (Figure 6(a-0.0)), showing a similar distribution to that in Figure 5(a-0.0). However, the number of RBS was much greater and the dimension of RBS became smaller with the length of only about 5 μm after the treatment at 60 °C (Figure 6(b-0.0), black circles) compared to that obtained after the treatment at 80 °C (Figure 5(b-0.0)). After the low content (0.5 and 1.0 wt %) of SiO_2_ nanoparticles were added in PDMS coating, RBS disappeared, and TPS appeared (Figure 6(a-0.5) and (a-1.0)), showing the same morphology as that obtained after the treatment at 80 °C (Figure 5(a-0.5) and (a-1.0)).

The number of TPS increased, but the dispersion of TPS showed no significant change compared to that of RBS (Figure 6(a-0.0)). Meanwhile, the dimension of TPS became smaller with a homogeneous distribution close to 1 μm (red dash circles in AFM images, Figure 6(b-0.5) and (b-1.0)) compared to that of RBS (Figure 6(b-0.0)). After high contents (2.0 wt %) of SiO_2_ nanoparticles were added into PDMS coating (Figure 6(a-2.0) and (b-2.0)), lots of NAS with homogeneous distribution were found, similar to that in Figure 5(a-2.0) and (b-2.0).

**Figure 7:**
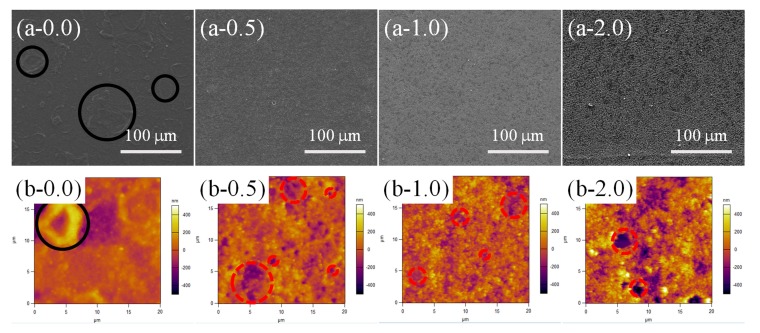
SEM (**a-*x***) and high-magnification AFM (**b-*x***) images for samples with different contents (***x***) of SiO_2_ at 60 °C-95% RH treatment.

Moreover, the effect of a higher RH on the surface morphology evaluation of samples with different contents of SiO_2_ (0.0, 0.5, 1.0 and 2.0 wt %) was also investigated at a lower temperature (60 °C for 90 min under 95% RH) (Figure 7). Compared with the samples in Figure 5(a-0.0) and Figure 6(a-0.0), a large number of RBS could be found on the entire surface of PPXC film with pure PDMS (Figure 7(a-0.0)). However, the number of RBS obviously decreased and the dimension of some RBS with a severely inhomogeneous distribution became as long as about 45 μm (or near 100 μm) (black circles in Figure 7(a-0.0) and (b-0.0)). For the coating with the low content (0.5 and 1.0 wt %) of SiO_2_ nanoparticles, RBS disappeared, and TPS occurred. The number of TPS shows little change (Figure 7(a-0.5) and (a-1.0)), showing the same morphology as the samples treated at 55% RH (Figure 6(a-0.5) and (a-1.0)). However, the dispersion and dimension of TPS increased a little (red dash circles in AFM images, Figure 7(b-0.5) and (b-1.0)) due to the appearance of some large-scale TPS compared to that in Figure 6(b-0.5) and (b-1.0). After high contents (2.0 wt %) of SiO_2_ nanoparticles were added in PDMS coating (Figure 7(a-2.0) and (b-2.0)), lots of NAS still could be found, but there were still a few TPS, shown as red dash circles in Figure 7(b-2.0) compared to the samples in Figure 5(b-2.0) and Figure 6(b-2.0). The TPS should be attributed to the higher RH adopted in the sample treatment at 60 °C-95% RH.

**Figure 8:**
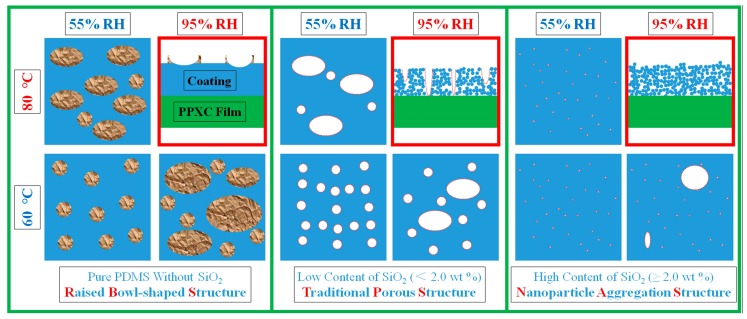
Morphology evolution scheme of the samples with different contents of SiO_2_, temperature and RH treatment conditions. Upper right schemes in red squares illustrate the cross-section morphologies of samples for raised bowl-shaped structures (RBS), traditional porous structures (TPS) and nanoparticle aggregation structures (NAS); schemes in upper left, lower left, and lower right illustrate the surface morphologies of samples with 80 °C-55% RH treatment, 60 °C-55% RH treatment, and 60 °C-95% RH treatment, respectively.

To clearly reveal the mechanism of surface morphology evolution, the effects of cross-linking reaction temperature (upper row for 80 °C and lower row for 60 °C), RH (left column for 55% RH and right column for 95% RH), and content of SiO_2_ (left column for pure PDMS without SiO_2_, middle column for PDMS with a low content of SiO_2_, and right column for PDMS with a high content of SiO_2_) are shown in Figure 8. Based on the results in Figure 5, Figure 6 and Figure 7, the conclusions can be drawn below.

Three different types of surface patterns (RBS, TPS, and NAS) with an area over 200 μm × 200 μm were generated on PPXC film mainly by controlling the content of SiO_2_ nanoparticles. Firstly, as for the coating on PPXC film without SiO_2_ nanoparticles, only RBS could be found on the surface even at different cross-linking reaction temperatures and under different RH treatment conditions. It should be attributed to the repellence of PDMS molecular chains to condensed water droplets; the PDMS molecular chains migrated and finally cured at the three-phase contact line (the interface of coating phase, condensed water droplets phase, and air phase) to form typical RBS after the evaporation of condensed water droplets, as shown in the upper right image of the left column in Figure 8. Secondly, as for coatings with low contents of SiO_2_ nanoparticles (between zero and 2.0 wt %) shown in the middle column of Figure 8, RBS disappeared but TPS occurred under different cross-linking reaction temperatures and RH treatment conditions. The changed phenomenon might be interpreted as follows. The introduction of SiO_2_ nanoparticles greatly hindered the migration of PDMS molecular chains to the three-phase contact line. Nonetheless, PDMS molecular chains underneath condensed water droplets could still migrate to SiO_2_ nanoparticle aggregations due to the repellence to the sinking condensed water droplets. Therefore, many pores were formed with the evaporation of condensed water droplets, similarly with the formation process of pores via the traditional “Breath Figures” method. Thirdly, with the increase of SiO_2_ nanoparticles, TPS were gradually transformed into NAS and there were almost no TPS when the content increased to no less than 2.0 wt % (the right column in Figure 8). Condensed water droplets hardly sank into the large number of aggregated SiO_2_ nanoparticles due to the lower movability of nanoparticles than PDMS molecular chains. These three typical physical structures can be further demonstrated on sample treated by a high thermal cross-linking temperature (80 °C) and RH treatment condition (95%) shown in Figure S1 in Supplementary Materials. There were still only RBS on the sample without SiO_2_ nanoparticles (Figure S1(a-0.0) and (b-0.0), black circles). TPS could be found with a low content of SiO_2_ nanoparticles (between zero and 2.0 wt %, red dash circles) (Figure S1(a-0.5), (a-1.0) and (b-0.5), (b-1.0)). Meanwhile, TPS were gradually changed to NAS and there were almost no TPS when the content of SiO_2_ nanoparticles increased above 2.0 wt % (Figure S1(a-2.0) and (b-2.0)).

Under the same content of SiO_2_ nanoparticles, the number, distribution, and dimension of RBS showed similar variations with TPS and these variations could be tuned by changing thermal cross-linking temperature and RH treatment condition. At a higher temperature and under the same RH treatment conditions, the faster thermal curing of PDMS could not provide enough time for the formation of a stable RH condition to generate a large number of homogeneous condensed water droplets on the coating surface. Meanwhile, water vapor had more energy to show a more drastic Brownian Motion, which resulted in the formation of larger condensed water droplets with a decrease in the regularity. Therefore, the number was smaller and the distribution was less homogeneous. The dimension was larger for RBS and TPS at 80 °C than that at 60 °C. Furthermore, compared to the lower RH condition (55%), the higher RH condition (95%) with more water vapor also resulted in the formation of larger condensed water droplets and a decrease in the number and regularity of RBS and TPS. We compared the samples (Figure S1) obtained with a higher thermal cross-linking temperature (80 °C) and higher RH treatment condition (95%) with the samples (Figure 5, Figure 6 and Figure 7) obtained with a lower thermal cross-linking temperature (60 °C) and/or lower RH treatment condition (55%) and found similar variations in RBS and TPS: the number of RBS or TPS became smaller; the distribution was less homogeneous, the dimension was increased.

Moreover, the number, distribution and dimension of TPS were affected by the content of SiO_2_ nanoparticles. The number decreased and the distribution became irregular. The dimension became smaller with the increase in the content of SiO_2_ nanoparticles (Figure 5(a-0.5)–(a-1.0), Figure 6(a-0.5)–(a-1.0), Figure 7(a-0.5)–(a-1.0) and Figure S1(a-0.5)–(a-1.0)). If the content of SiO_2_ nanoparticles reached 2.0 wt %, TPS nearly disappeared and were transformed into NAS because the migration of PDMS molecular chains had been seriously hindered by the high content of SiO_2_ nanoparticles. Therefore, the thermal cross-linking temperature and RH treatment condition showed little effect on NAS, which seemed to be only controlled by the content of SiO_2_ nanoparticles (the right column in Figure 8).

### 3.3. Surface Wettability of PDMS/SiO_2_ Coating on PPXC Film under Different Treatment Conditions

**Figure 9:**
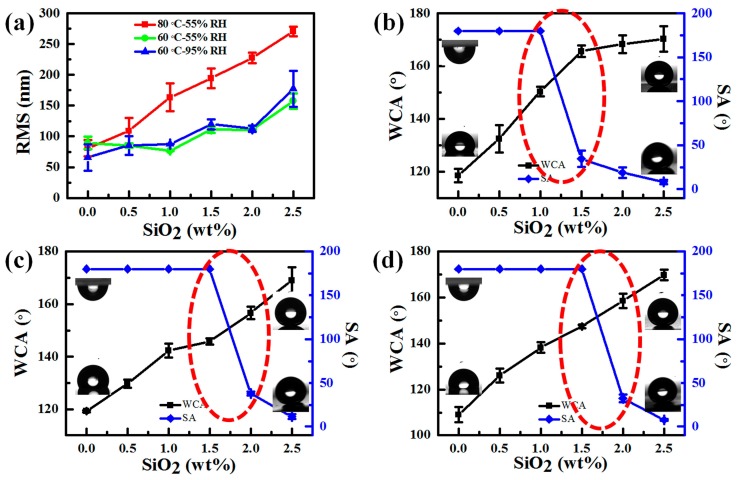
(**a**) Statistic surface roughness (RMS) on the samples with different contents of SiO_2_ under different RH treatment conditions (80 °C-55% RH treatment (red square), at 60 °C-55% RH treatment (green circle), and at 60 °C-95% RH treatment (blue triangular)); WCA and SA on the samples with different contents of SiO_2_ under different RH treatment conditions (80 °C-55% RH treatment (**b**); 60 °C-55% RH treatment (**c**); at 60 °C-95% RH treatment (**d**)). Profiles in the lower left and upper right in Figure 9b–d show the behaviors of static water droplets on the surface. Profiles in the upper left indicate that the water droplets cannot slide off the surface even after 180° reversal of the surface, but profiles in the lower right indicate that the water droplets easily slide off the surface within 10° SA. Water droplets still cannot slide off the surface with a “Wenzel” state shown in the red dash circles.

**Figure 10:**
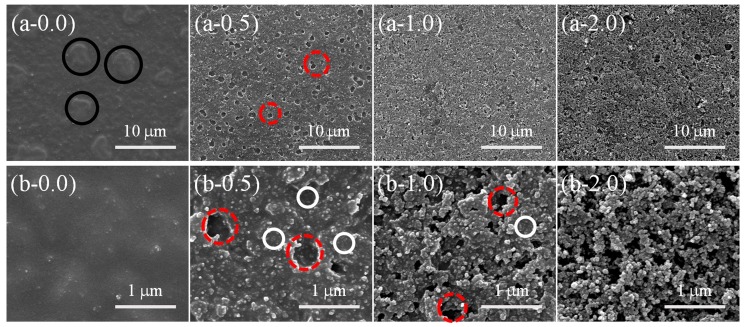
Surface micro-scale (SEM, **a-*x***) and nano-scale (SEM, **b-*x***) morphologies of the samples with different contents (***x***) of SiO_2_ at 60 °C-55% RH.

According to previous studies, the surface wettability was usually determined by the rough physical structures and low surface energy chemical compositions. As the materials used in this study (both PDMS and hydrophobic SiO_2_ shown in Figure 4) were hydrophobic under different treatment conditions, the evolution of surface morphologies (Figure 5, Figure 6 and Figure 7) should be responsible for the variations in surface wettability (Figure 9). RMS calculated from AFM images is shown in Figure 9a. The surface roughness increased monotonously and rapidly with the increase in the content of SiO_2_ nanoparticles for the samples treated at 80 °C, but RMS showed a tiered growth for the samples treated at 60 °C (similar RMS of 85 nm for the samples with zero, 0.5, and 1.0 wt % SiO_2_; similar RMS of 110 nm for the samples with 1.5 and 2.0 wt % SiO_2_; and similar RMS above 150 nm for the samples with 2.5 wt % SiO_2_). This special variation of RMS was ascribed to a faster thermal curing of PDMS at 80 °C than at 60 °C, so the surface physical structures were quickly immobilized without enough time to transform to homogeneous structures. The RMS variation of the samples treated at 60 °C-55% RH and 60 °C-95% RH indicated that RH had little effect on the statistic surface roughness but showed obvious effects on the number, distribution, and dimension of surface structures (Figure 5, Figure 6, Figure 7 and Figure 8 and Figure S1). Due to the increase in RMS, WCA also increased gradually with the increase in the content of SiO_2_ nanoparticles (Figure 9b–d). As for the samples with RBS (pure PDMS without SiO_2_), WCA (Figure 9b,c) was about 118° and showed little increase compared to that on smooth PDMS [48] due to the increased roughness caused by RBS patterns (Figure 5 or Figure 6). Furthermore, WCA in Figure 9d was only about 108° for the sample treated at 60 °C-95% RH due to the much smoother RBS with a lower RMS (Figure 7, about 65 nm) compared to that on the samples treated at 80 °C-55% RH and 60 °C-55% RH (Figure 5 and Figure 6, about 89 nm). Water droplets could not slide off all the samples with RBS even after 180° reversal of the samples (the upper left profiles in Figure 9b–d), indicating that water droplets on the surface were at a typical “Wenzel” state [49]. This should result from a lack of enough nano-scale roughness and the existence of only some micro-scale RBS (black circles in Figure 10, Figures S2 and S3) on the surface. Moreover, water droplets on the samples with TPS also could not slide off the surface (the left of red dash circles in Figure 9b–d). As shown in the surface morphologies of the samples treated at 60 °C-55% RH in Figure 10(a-0.5), (b-0.5), (a-1.0), and (b-1.0) and the samples treated at 80 °C-55% RH and 60 °C-95% RH in Figures S2 and S3, micro-scale TPS (red dash circles) and nano-scale roughness based on SiO_2_ nanoparticle aggregations were increased, thus improving the surface hydrophobicity. However, water droplets were pinned on these samples with a WCA lower than 150° for two reasons. Firstly, the low nano-scale roughness was caused by the too-low content of SiO_2_ nanoparticles (0.5 or 1.0 wt %) and the submersion of some SiO_2_ nanoparticles into PDMS (white circles in Figure 10, Figure S2 and Figure S3). Secondly, the capillary effect of the pores from TPS and the gaps formed between SiO_2_ nanoparticle aggregations shown in red dash circles in Figure 10, Figure S2, and Figure S3 led to the phenomenon of water droplets.

When WCA on the samples was higher than 150° (the right of red dash circles in Figure 9b–d), water droplets began to slide off the surface with an SA less than 40°, showing a transition of water droplets from a “Wenzel” state to a “Cassie” state [49,50,51]. Samples in the left area of the circles were in a “Wenzel” state because water droplets could not slide off the surface even after 180° reversal of the surface, and samples in the right area of the circles were in a “Cassie” state because water droplets began to slide off the surface with the help of the release of air bubbles underneath the droplets. This transition is consistent with the increase of surface RMS (Figure 9a). The samples treated at 60 °C-55% RH and 60 °C-95% RH showed a typical tiered growth of RMS and a sharp increase under 1.5 wt % of SiO_2_ nanoparticles, displaying a WCA close to 150°. The transition started under the conditions of 1.5 wt % of SiO_2_ nanoparticles and a WCA close to 150°. The special tiered growth of RMS should be interpreted as follows. Curing time required at 60 °C was longer than that at 80 °C so that the migration of PDMS could achieve the more homogeneous morphologies. When the content of SiO_2_ nanoparticles reached 2.5 wt %, in the sample treated at 80 °C-55% RH, RMS continued to increase to about 270 nm; in the samples treated at 60 °C-55% RH and 60 °C-95% RH (Figure 9a), RMS sharply increased, thus resulting in a further increase of WCA and water droplets easily rolling off the surface with an SA of only about 8°. All the samples showed a typical “Cassie” state (Figure 9b–d). The changes should be ascribed to the special NAS similar to the sample containing 2.0 wt % of SiO_2_ nanoparticles (Figure 10(a-2.0) and (b-2.0)) and our previous studies.

**Figure 11:**
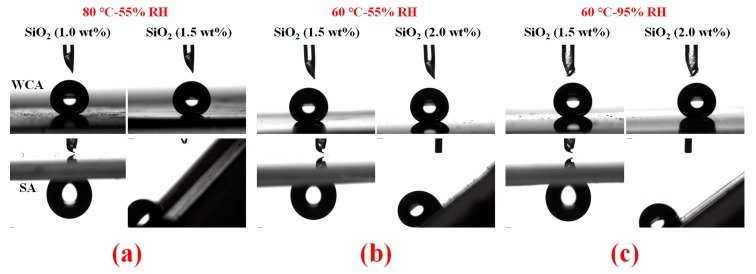
Profiles of water droplets for WCA (upper row) and SA (lower row) on different samples after being left in room conditions for about 6 months with different contents (left and right columns) of SiO_2_ at 80 °C-55% RH (**a**); 60 °C-55% RH (**b**); and 60 °C-95% RH (**c**).

Moreover, the precise transitions of water droplets from a “Wenzel” state to a “Cassie” state for samples treated at different conditions are shown in red dash circles in Figure 9b–d. Meanwhile, the profiles of water droplets for WCA and SA on different samples after being left in room conditions for about 6 months were shown in Figure 11. All the WCAs were close to 150° and water droplets could not slide off the surfaces even after 180° reversal of the samples if the samples were in a “Wenzel” state shown in the left columns of Figure 11a–c. However, water droplets began to slide off the surfaces due to the repellency of air bubbles underneath the water droplets if the samples were in a “Cassie” state, as shown in the right columns of Figure 11a–c. WCAs and the sliding behavior of water droplets on these samples showed little change compared with the as-prepared samples in Figure 9. Therefore, these samples showed a stable hydrophobicity or superhydrophobicity. This stability should be attributed to the complete cross-linking of PDMS after thermal treatment as demonstrated in Figure 2, Figure 3 and Figure 4. Thus, these structures, RBS, TPS, and NAS, were achieved with one-step treatment within 90 min.

**Figure 12:**
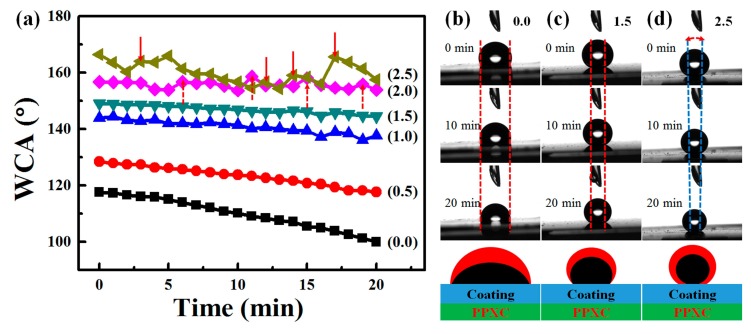
The evolution of WCA with time after deposition of the water droplets on samples with different contents of SiO_2_ at 60 °C-55% RH (**a**); the profiles of water droplets on sample with 0.0 wt % (**b**); 1.5 wt % (**c**); and 2.5 wt % SiO_2_ (**d**); with 0 min, 10 min, and 20 min. The schemes in Figure 12b–d show the evolution of water droplets before (red) and after (black) evaporation. The red and blue dash lines in Figure 12b–d showed the edges of the three-phase contact lines during the evaporation of water droplets. The gradually decreased length of the three-phase contact line in Figure 12d was illustrated by the red dash arc double arrows and the scheme.

The temporal changes of WCA on hydrophobic or superhydrophobic surfaces usually show only a little change, for example, less than 10° decrease in 25 h for samples in the reference [19], and less than 5° change in 30 days for superhydrophobic samples in previous publications [47]. In consideration of different kinds of structures in this manuscript, the temporal changes of WCA on samples with RBS, TPS, and NAS are shown in Figure 12. However, WCAs on RBS (0.0 wt % SiO_2_) in Figure 12a,b decreased about 17° with 20 min. This obvious decrease should be attributed to the firm adhesion of water droplets on RBS, and the three-phase contact line showed almost little change during the evaporation of water droplets. As for WCA on RBS (1.5 wt % SiO_2_) in Figure 12a,c, it showed only about 5° change, which should result from the shorter length of the three-phase contact line with a higher initial WCA. Meanwhile, the WCAs within 20 min for samples with lower contents of SiO_2_ (less than 2.0 wt %) showed a steady decrease, which should be due to the samples in a “Wenzel” state and the firm adhesion of water droplets on RBS and TPS. When the contents of SiO_2_ increased to 2.0 wt % and 2.5 wt %, WCA showed wavy change, which should be due to the samples in a “Cassie” state. With a “Cassie” state, air bubbles underneath the water droplets would escape from the edge of the three-phase contact lines during the evaporation of water droplets. The release of air bubbles would shorten the length of the three-phase contact line (blue dash lines in Figure 12d) and result in an increased WCA shown as red dash upward arrows (2.0 wt %) and red downward arrows (2.5 wt %) in Figure 12a.

## 4. Conclusions

In summary, PDMS could be well thermally cured at a temperature of 60 °C or higher for 90 min. Meanwhile, the content of SiO_2_ nanoparticles and the relative humidity (55% and 95%) showed little effect on the complete curing of PDMS at 60 °C or higher temperature. However, among the samples treated at different temperatures and RH conditions, the cross-linking reaction process was different and resulted in different surface morphologies. By changing the contents of SiO_2_ nanoparticles, thermal cross-linking temperatures, or RH treatment conditions, three different types of large-scale surface patterns (RBS, TPS, and NAS) were obtained on PPXC film. Meanwhile, the number, distribution, and dimension of these three pattern structures were tuned by changing the experimental conditions. With the evolution of surface patterns, the surface wettability was changed from weak hydrophobicity to superhydrophobicity and the WCA increased from 84° to 168°. A typical transition of water droplets from a “Wenzel” state to a “Cassie” state on the surface was observed. The PPXC film with different surface patterns of 200 μm × 200 μm and the improved surface hydrophobicity showed wide application potentials in self-cleaning, electronic engineering, micro-contact printing, cell biology, tissue engineering, and so on.

## Supplementary Materials

The following are available online at http://www.mdpi.com/1996-1944/11/4/486/s1, Figure S1: SEM (a-*x*) and high-magnification SEM (b-*x*) images for the samples with different contents (*x*) of SiO_2_ at 80 °C-95% RH treatment, Figure S2: Surface micro-scale (SEM, a-*x*) and nano-scale (SEM, b-*x*) morphologies of the samples with different contents (*x*) of SiO_2_ at 80 °C-55% RH, Figure S3: Surface micro-scale (SEM, a-*x*) and nano-scale (SEM, b-*x*) morphologies of the samples with different contents (*x*) of SiO_2_ at 60 °C-95% RH.
