# Supplementary material for: Patternable Poly(chloro-p-xylylene) Film with Tunable Surface Wettability Prepared by Temperature and Humidity Treatment on a Polydimethylsiloxane/Silica Coating
Source: Materials (Basel). 2018 Mar 23;11(4):486. doi: 10.3390/ma11040486 (PMC5951332; doi:10.3390/ma11040486)
Supplement: Supplementary file 1 [file materials-11-00486-s001.pdf]

materials

MDPI

Article

Patternable Poly(chloro-p-xylylene) Film with Tunable Surface Wettability Prepared by Temperature and Humidity Treatment on a Polydimethylsiloxane/Silica Coating

Yonglian Yu[1,2], Hong Shao[2], Zhoukun He[2,*], Changyu Tang[2], Jian Yang[2], Yongsheng Li[2,3], Cong Wang[2], Xiuyun Li[1,*], Maobing Shuai[3], Jun Mei[2]

1 State Key Laboratory Cultivation Base for Nonmetal Composites and Functional Materials, School of Materials Science and Engineering, Southwest University of Science and Technology, Mianyang, 621010, China; 664287931@qq.com (Y. Yu)

2 Chengdu Green Energy and Green Manufacturing Technology R&D Center, Chengdu Development Center of Science and Technology, China Academy of Engineering Physics, Chengdu, 610207, China; 1048214406@qq.com (H. Shao); sugarchangyu@163.com (C. Tang); 479775804@qq.com (J. Yang); yongsli718@163.com (Y. Li); congwang_polymer@163.com (C. Wang); meijun12@126.com (J. Mei)

3 Science and Technology on Surface Physics and Chemistry Laboratory, Mianyang, 621907, China; shuaimb@sina.com (M. Shuai)

* Correspondence: hezhoukunhe@163.com (Z. He); Tel.: +86-822-440-7693; lixiuyun_2002@163.com (X. Li); Tel.: +86-390-901-9537

Received: date; Accepted: date; Published: date

Abstract: Poly(chloro-p-xylylene) (PPXC) film has a water contact angle (WCA) of only about 84°. It is necessary to improve its hydrophobicity for the prevention of liquid water droplets from corroding or electrically shorting metallic circuits of semiconductor devices, sensors, microelectronics and so on. Herein, we reported a facile approach to improve its surface hydrophobicity by varying surface pattern structures under different temperature and relative humidity (RH) conditions on a thermal curable polydimethylsiloxane (PDMS) and hydrophobic silica (SiO_2) nanoparticle coating. Three distinct large-scale surface patterns were obtained mainly depending on the contents of SiO_2 nanoparticles. The regularity of patterns was mainly controlled by the temperature and RH conditions. By changing the pattern structures, the surface wettability of PPXC film could be improved and its WCA was increased from 84° to 168°, displaying a superhydrophobic state. Meanwhile, it could be observed that water droplets on PPXC film with superhydrophobicity were transited from a "Wenzel" state to a "Cassie" state. The PPXC film with different surface patterns of 200 μm × 200 μm and the improved surface hydrophobicity showed wide application potentials in self-cleaning, electronic engineering, micro-contact printing, cell biology, and tissue engineering.

Keywords: Poly(chloro-p-xylylene); Surface wettability; Polydimethylsiloxane; Silica nanoparticles; Superhydrophobic coating

1. Supplementary Materials

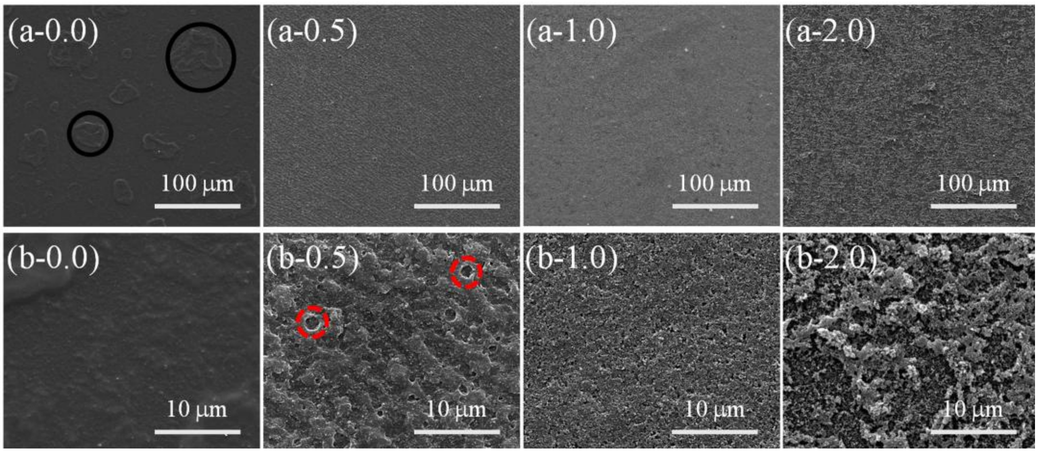

Figure S1. SEM **(a-x)** and high-magnification SEM **(b-x)** images for the samples with different contents **(x)** of SiO_2 at 80 °C-95% RH treatment.

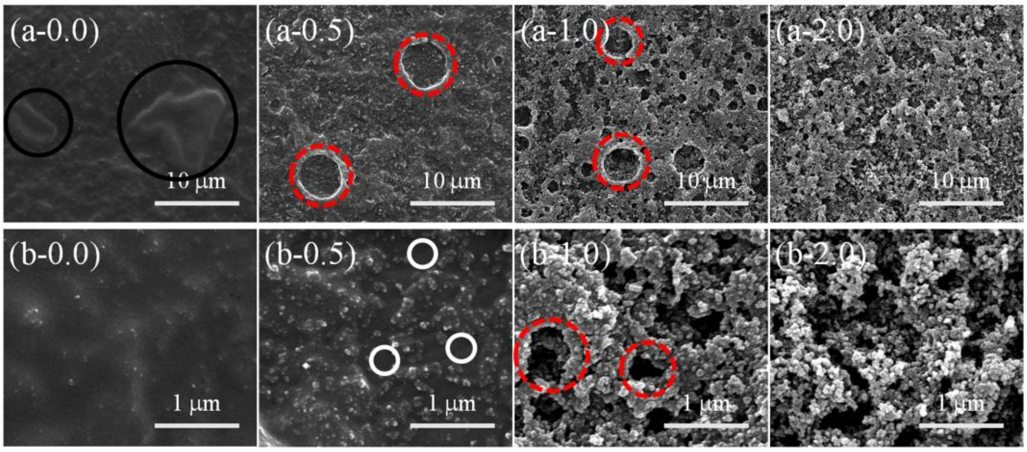

Figure S2. Surface micro-scale (SEM, **a-x**) and nano-scale (SEM, **b-x**) morphologies of the samples with different contents (**x**) of SiO_2 at 80 °C-55% RH.

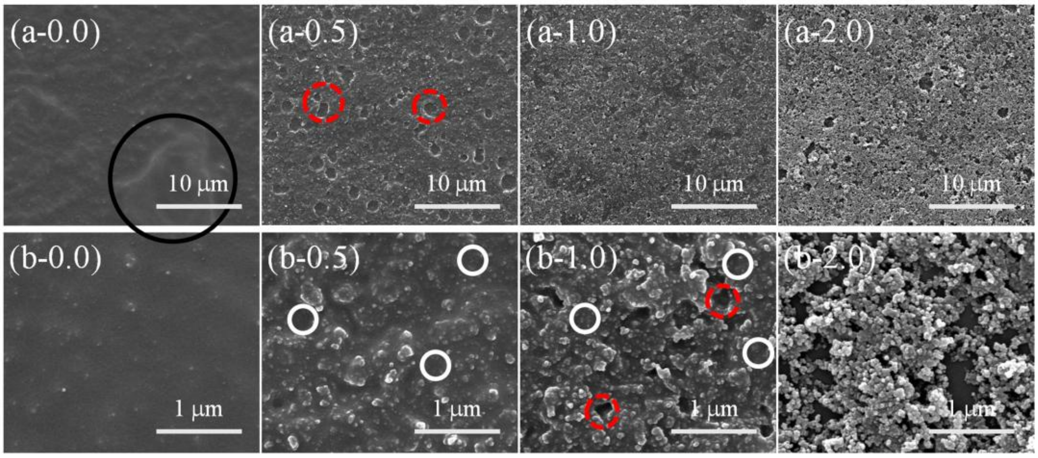

Figure S3. Surface micro-scale (SEM, **a-x**) and nano-scale (SEM, **b-x**) morphologies of the samples with different contents (**x**) of SiO_2 at 60 °C-95% RH.

© 2018 by the authors. Licensee MDPI, Basel, Switzerland. This article is an open access article distributed under the terms and conditions of the Creative Commons Attribution (CC BY) license (http://creativecommons.org/licenses/by/4.0/).
